# A Case of Aneurism of the Brachial Artery

**Published:** 1871-01

**Authors:** C. B. Kibler

**Affiliations:** Corry, Pa.


ART. IV.—*A Case of Aneurism of the Brachial Artery.* By C. B. KIBLER, Corry, Pa.

Was called to see John D——, August 17th, 1870, aged 29 years. He was suffering with intense pain in his right hand, wrist, forearm and arm.

In the upper third of the brachial artery, presented a pulsating tumor, about the size of a large orange, which was first noticed about six weeks prior. The supposed cause was his swinging on a horizontal bar. The blowing sound, together with the cessation of pulsation of the tumor by pressure upon the artery above it, were almost pathognomonic signs of aneurism, and made diagnosis accordingly.

On August 25th, 1870, Dr. Palmer, assisted by Dr. Faulkner and myself, ligated the axillary artery in its lower third. Everything moved off pleasantly in the case up to the second day of September, when a messenger came hurriedly to the office and said the patient was bleeding to death. When I arrived at the bed side of the patient, I found the blood spirting from the point where the ligature had been applied. Caused digital compression of the subclavian artery to be made, sent for another physician, and in the meantime commenced the administration of chloroform, so no time should be lost. The physician sent for soon arrived and continued the administration of chloroform, while I ligated the axillary artery in the upper third of its course.

Everything now went on nicely—in five weeks time the ligature came away, and the wound was entirely healed. He yet has but limited use of his hand and forearm, with no pulsation of the radial artery.

